# Simultaneous Calibration: A Joint Optimization Approach for Multiple Kinect and External Cameras

**DOI:** 10.3390/s17071491

**Published:** 2017-06-24

**Authors:** Yajie Liao, Ying Sun, Gongfa Li, Jianyi Kong, Guozhang Jiang, Du Jiang, Haibin Cai, Zhaojie Ju, Hui Yu, Honghai Liu

**Affiliations:** 1 Key Laboratory of Metallurgical Equipment and Control Technology, Wuhan University of Science and Technology, Ministry of Education, Wuhan 430081, China; liaoyajie123@126.com (Y.L.); sunying@wust.edu.cn (Y.S.); kongjianyi@wust.edu.cn (J.K.); whjgz@wust.edu.cn (G.J.); 2 Hubei Key Laboratory of Mechanical Transmission and Manufacturing Engineering, Wuhan University of Science and Technology, Wuhan 430081, China; jiangdu1231@163.com; 3 School of Computing, University of Portsmouth, Portsmouth PO1 3HE, UK; haibin.cai@port.ac.uk (H.C.); zhaojie.ju@port.ac.uk (Z.J.); hui.yu@port.ac.uk (H.Y.); honghai.liu@port.ac.uk (H.L.)

**Keywords:** joint calibration, Kinect, external camera, depth camera

## Abstract

Camera calibration is a crucial problem in many applications, such as 3D reconstruction, structure from motion, object tracking and face alignment. Numerous methods have been proposed to solve the above problem with good performance in the last few decades. However, few methods are targeted at joint calibration of multi-sensors (more than four devices), which normally is a practical issue in the real-time systems. In this paper, we propose a novel method and a corresponding workflow framework to simultaneously calibrate relative poses of a Kinect and three external cameras. By optimizing the final cost function and adding corresponding weights to the external cameras in different locations, an effective joint calibration of multiple devices is constructed. Furthermore, the method is tested in a practical platform, and experiment results show that the proposed joint calibration method can achieve a satisfactory performance in a project real-time system and its accuracy is higher than the manufacturer’s calibration.

## 1. Introduction

Camera calibration is a process of estimating intrinsic parameters (such as focal length, principal point and lens distortion) and extrinsic parameters (such as rotation and translation) of camera (including color camera and depth camera) [1]. It has been widely used in computer/machine vision, and it makes the measurement of distances in the real world from their projections on the image plane possible [2]. Thus, with the continuous development of computer/machine vision, the camera calibration has been widely applied in 3D reconstruction [3,4], structure from motion [5], object tracking [6,7,8] and gesture recognition [9,10], etc.

On 4 November 2010, with the launch of low-cost Microsoft Kinect sensors (Los Angeles, CA, USA) (the image capture device of the Kinect includes a color camera and a depth sensor which consists of an infrared (IR) projector combined with an IR camera), 3D depth cameras are increasingly attracting researchers due to their versatile applications in computer vision [11]. However, it is well known that Kinect intrinsics vary from device to device, which leads to the fact that the factory presets are not accurate enough for many applications [12]. To deal with the above issue, Burrus [13] presented a basic Kinect calibration algorithms by using camera calibration process based on OpenCV. However, it only calibrated the intrinsic parameters of the infrared camera. On the other hand, Hirotake et al. [14] tried to independently calibrate the intrinsic parameters of the depth sensor and color camera, and then register both in a common reference frame. Herrera et al. [15] proposed a color camera calibration method with high-precision to assist the Kinect calibration. Their approach can achieve a high accuracy. In addition, Zhang et al. [16] augmented Herrera’s work with correspondences matching between the color and depth images, but they did not address distortions in the depth values. Smisek et al. [17] first considered the distortions in the projection and the depth estimation. After calibrating the internal and external parameters of the device, the depth distortion of each pixel was estimated by averaging the metric error. Moreover, focusing on the distortion for depth maps, Herrera et al. [18] proposed a joint depth and color camera calibration, and used the Lambert W function to solve the disparity distortion model. This process improved the calibration accuracy and corrected the depth distortion. However, their methods were generally limited to a single external camera, and could not be effectively employed with multiple devices. After that, Carolina et al. [19] and Guo et al. [20] improved the performance on the basis of the Herrera’s work: Carolina et al. proposed a metric constraint and used an open-loop post-processing step; Guo et al. simplified the disparity distortion model with the Taylor formula. Both of them improved the calibration speed and reduced the amount of input pictures. Han et al. [21] used two Kinects to form up a depth camera network, and accordingly achieved a fast and robust camera calibration process. Nonetheless, they still did not consider a joint calibration for multiple external cameras.

Current research only focuses on the calibration of a single external camera instead of the calibration of multiple external cameras. To this end, this paper aims at filling this gap. This paper introduces a novel method and a corresponding workflow framework, which can simultaneously calibrate a Kinect, three external cameras, and their relative positions. By optimizing the final cost function and adding corresponding weights to the external cameras in different locations, the joint calibration of the depth sensor in Kinect and multiple external high- resolution color cameras is realized. The paper is organized as follows: Section 2 introduces the calibration model; Section 3 proposes the approach to jointly calibrate the multiple sensors; Section 4 discusses the comparative experimental results and the conclusions are presented in the final session.

## 2. Calibration Model

### 2.1. Color Camera Projection Model

In this paper, the intrinsic model of the color camera is similar to that in [22], which is described by a pinhole model with radial and tangential distortion coefficients. It is assumed that the color camera coordinate is ${Χ}_{C}={\left[x_{c},y_{c},z_{c}\right]}^{T}$, and it can be normalized as $X_{n}={\left[x_{n},y_{n}\right]}^{T}={\left[x_{c}/z_{c},y_{c}/z_{c}\right]}^{T}$. In the pinhole model, a straight line may bend due to the effect of radial distortion [23], which can be solved by the following formula:

(1) $$\begin{array}{l} x_{cor}=x_{n}\left(1+k_{1}r^{2}+k_{2}r^{4}+k_{3}r^{6}\right)\\ y_{cor}=y_{n}\left(1+k_{1}r^{2}+k_{2}r^{4}+k_{3}r^{6}\right) \end{array}$$

Similarly, tangential distortion happens when the camera lens is not perfectly parallel to the image plane, which causes some areas of the image to look closer than expected [24]. It can be solved by the following formula:

(2) $$\begin{array}{l} x_{cor}=x_{n}+\left[2p_{1}x_{n}y_{n}+p_{2}\left(r^{2}+2x_{n}^{2}\right)\right]\\ y_{cor}=y_{n}+\left[p_{1}\left(r^{2}+2y_{n}^{2}\right)+2p_{2}x_{n}y_{n}\right] \end{array}$$

where $r^{2}=x_{n}^{2}+y_{n}^{2}$, $\left(x_{cor},y_{cor}\right)$ represents the corrected coordinate point. $k_{1}$, $k_{2}$, $k_{3}$ and $p_{1}$, $p_{2}$ are the radial and tangential distortion coefficients, respectively [25]. Therefore, $K=\left[k_{1},k_{2},p_{1},p_{2},k_{3}\right]$ is used to represent the distortion coefficients. In addition, $K_{c}=\left[k_{c1},k_{c2},p_{c1},p_{c2},k_{c3}\right]$ and $K_{d}=\left[k_{d1},k_{d2},p_{d1},p_{d2},k_{d3}\right]$ represent the distortion coefficients of the color and depth cameras, respectively.

Then, the image coordinates can be obtained by:

(3) $$\left[\begin{array}{c} u\\ v \end{array}\right]=\left[\begin{array}{cc} f_{x} & 0\\ 0 & f_{y} \end{array}\right]\left[\begin{array}{c} x_{cor}\\ y_{cor} \end{array}\right]+\left[\begin{array}{l} u_{0}\\ v_{0} \end{array}\right]$$

where $f=\left(f_{x},f_{y}\right)$ is the focal length and $P_{0}=\left(u_{0},v_{0}\right)$ is the principal point of the image coordinate P=(u,v). The same model can be applied to the color and external cameras [26]. In this paper, the subscript *c* and *d* are used to distinguish the same parameters for the color camera and the depth camera, respectively. For example, $f_{c}=\left(f_{cx},f_{cy}\right)$ represents the focal length of the color camera.

### 2.2. Depth Camera Intrinsic

The transformation relation between the depth camera coordinates and the depth image coordinates is similar to the model for the color camera. The distortion of the color camera is a forward model (i.e., from the world coordinates to the image coordinates), and for easy calculations, the geometric distortion of the depth camera uses the backward model [18] (i.e., from the image coordinates to the world coordinates). According to the imaging principle of the depth sensor, the relation between the obtained disparity value $d_{k}$ and the depth value $z_{k}$ can be expressed as:

(4) $$z_{k}=\frac{1}{c_{1}d_{k}+c_{0}}=\frac{1}{\frac{1}{f_{d}b}d_{k}+\frac{1}{z_{0}}}$$

where $z_{0}$ is the distance from the reference point to the reference plane, $f_{d}$ is the focal length of the depth camera, and *b* is the baseline length, which is the distance between the infrared camera and the laser emitter. $c_{1}=1/\left(f_{d}b\right)$ and $c_{0}=1/z_{0}$ are part of the intrinsic parameters of the depth camera that are required to be calibrated. If the measured value of disparity d is directly substituted into Equation (4) for calibration (i.e., the disparity distortion correction is not performed). The depth information in the observation process produces a fixed error that could be corrected by adding a spatially varying offset $Z_{\delta }$. It can effectively reduce the re-projection error [17], where the depth value $z_{kk}$ can be re-expressed as:

(5) $$z_{kk}=z_{k}+Z_{\delta }\left(u,v\right)$$

In order to improve the calibration accuracy, the method in [18] is used to directly correct the original disparity d. The method in [18] took the errors of all pixels from planes at several distances and normalized them. It can be found that the normalization error satisfies the exponential decay [19]. Therefore, a distortion model can be constructed to use an attenuated spatial offset to counteract the increasing disparity error. It can be expressed as:

(6) $$d_{k}=d+D_{\delta }\left(u,v\right)\exp\left(\alpha_{0}-\alpha_{1}d\right)$$

where d is the uncorrected disparity value obtained from Kinect, $D_{\delta }$ is used to eliminate the influence of the distortion, and it represents the spatial distortion related to each pixel. $\alpha_{0},\;\alpha_{1}$ represent the decay of the distortion effect, and $d_{k}$ is the corrected disparity value.

Equations (4) and (6) are used to calculate the disparity-to-depth transformation process, and the inverse of these equations can be used to calculate the re-projection error. According to the inverse of Equation (4), it is known that:

(7) $$d_{k}=\frac{1}{c_{1}z_{k}}-\frac{c_{0}}{c_{1}}$$

Equation (6) has an exponential relationship, so its inverse is much more complex than the inverse of Equation (4). Therefore, we can use Guo’s method that simplified Equation (6) by Taylor’s equation [20]:

(8) $$\begin{array}{ll} d_{k} & =d+D_{\delta }\left(u,v\right)\exp\left(\alpha_{0}-\alpha_{1}d\right)\\ & \approx d+D_{\delta }\left(u,v\right)\left(1+\alpha_{0}-\alpha_{1}d\right) \end{array}$$

Hence,

(9) $$d=\frac{d_{k}-D_{\delta }-D_{\delta }\alpha_{0}}{1-D_{\delta }\alpha_{1}}$$

The model for the depth camera is described by $L_{d}=\left\{f_{d},P_{d0},K_{d},c_{0},c_{1},D_{\delta },\alpha_{0},\alpha_{1}\right\}$, where the first three represent internal parameters of depth camera, and the last five are used to transform disparity-to-depth values.

## 3. Joint Calibration for Multi-Sensors

**Figure 1:**
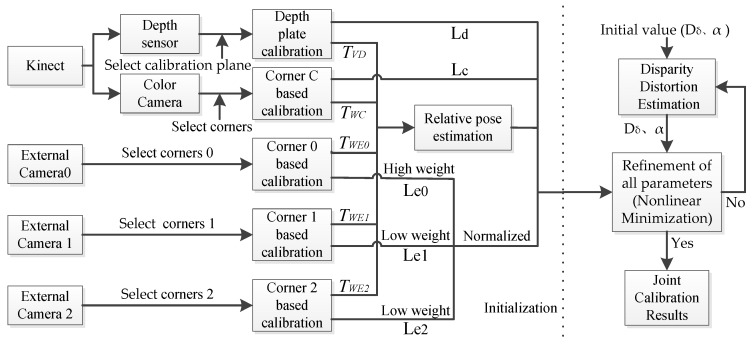
The illustration of work flow of the proposed method.

The block diagram of the proposed calibration method is presented in Figure 1. The proposed calibration method consists of three main consecutive steps: (1) selecting all the checkerboard corners by Zhang’s method [27] to initially estimate the intrinsic parameters of camera, and the four corners of the calibration plane are extracted in a depth map to initially estimate the intrinsic parameters of depth camera; (2) using Herrera’s method [18] to estimate the relative positions (extrinsic parameters) between the devices; and (3) initializing the disparity distortion parameters. Then, substituting all the parameters into the new proposed cost function and attaching different weights to iteratively calculate the nonlinear minimization. 

In the workflow framework, Step 1 and Step 2 contribute to the initialization of the parameters. They introduce the new parameters to the cost function in Step 3 for nonlinear minimization. In Step 3, when the disparity distortion function is calculated with the least squares method, the cost function of disparity distortion is the same as the corresponding intermediate term of the new cost function and does not interact with the other parameters. Therefore, after providing the corresponding initial value, the nonlinear minimization of the parameters can be achieved by iteratively calculating the new cost function. When all the parameters meet a predefined range, the joint calibration results can be output. Otherwise, it will continue to the next loop until the maximum number of iterations is reached.

### 3.1. Platform Setting and Preprocessing

**Figure 2:**
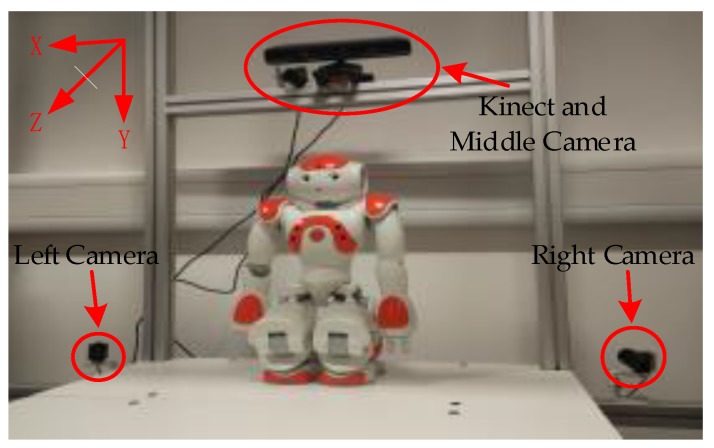
Relative positions among Kinect and three cameras on the framework.

The experimental platform with multiple sensors is shown in Figure 2. Kinect is located in front of children with Autism Spectrum Disorders (ASD), and the same place has an external color camera, which is called a Middle Camera (External Camera 0). Similarly, in the lower left corner and lower right corner are the other external color cameras, which are called Left Camera (External Camera 1) and Right Camera (External Camera 2), respectively. They are fixed on the same rigid platform and do not change the relative position during the course of the experiment, and the color camera of Kinect is set to coincide with the origin of the experimental frame coordinate system. At the same time, the direction of the experimental frame coordinate system is also shown in Figure 2.

**Figure 3:**
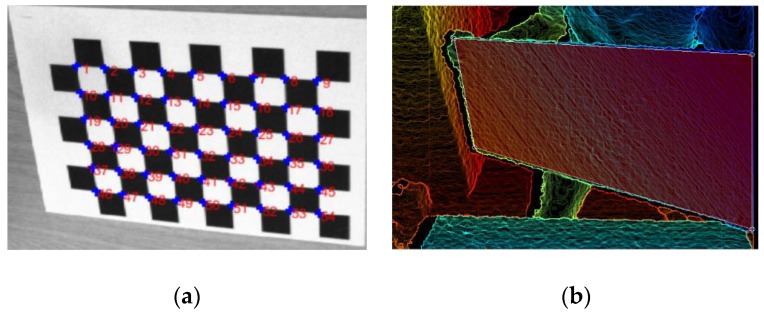
The key steps in the operation of the program. (**a**): the detection of corners—there are 54 corners of our checkerboard; (**b**): selected the four corners of the calibration plate—the manually selected plane coincides with the plane of the calibration plate.

In the process of selecting the checkerboard corners, Zhang’s method [27] is used to initialize the parameters of the color camera in Kinect and three external cameras. Using a standard checkerboard grid with a width of 0.025 m, and there are nine and six corner points in the *x*-axis and *y*-axis directions, respectively. The detection of corners is shown in Figure 3a. When the number of the input images is larger than three, the unique solution of Equation (3) can be found by Zhang’s method [27]. In this paper, in order to ensure the accuracy of the calibration results, when acquiring the image, three datasets are recorded at the distance of 0.8 m, 1.6 m and 2.4 m away from the camera frame plane. Each dataset is divided into five pictures, which include one picture of frontal plane, two pictures of the *x*-axis rotated plane and two pictures of the *y*-axis rotated plane. Generally speaking, the corners of the checkerboard cannot be displayed in the depth image, and we can only select four corners of the calibration plate in the depth image, as shown in Figure 3b. Although the accuracy of Kinect depth image is on the millimeter level, however, there is still a lot of noise in these corners. Consequently, the plane formed by the four selected corners can only be used to initially estimate the depth data of the calibration plate plane.

### 3.2. Relative Pose Estimation

**Figure 4:**
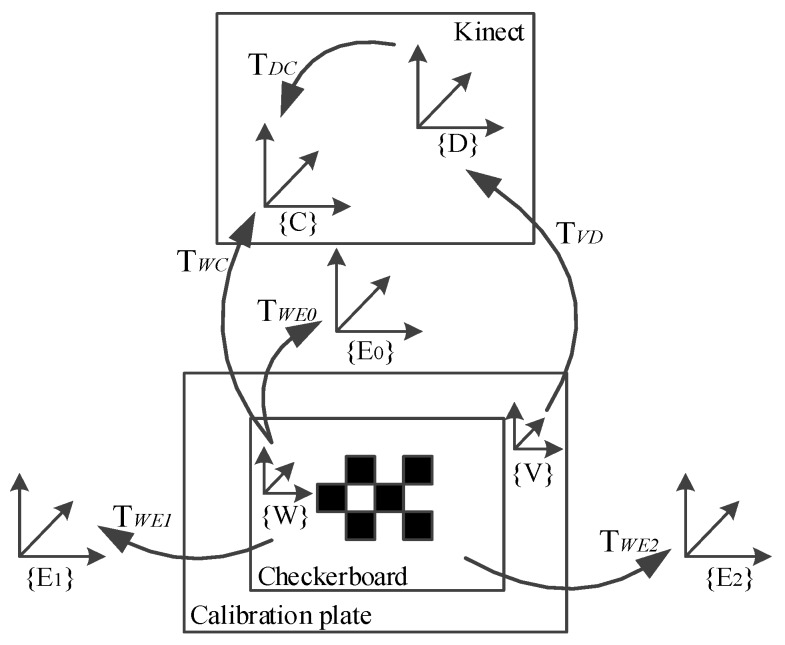
Reference frames and transformations. {D}, {C} and ({E0}, {E1}, {E2}) are the coordinate systems of depth, color, and external cameras, respectively.

In the relative position estimation, the color camera of Kinect is assumed to be the origin of the experimental frame coordinate system. All of the equipment is fixed on the same rigid frame during the whole experiment. All of the reference frames and transformations are illustrated in Figure 4. {D}, {C}, {W}, {V} and ({E0}, {E1}, {E2}) are the coordinate system of depth, color, checkerboard (world), calibration plate and external cameras, respectively. A point on a coordinate system can be transformed to another coordinate system by T = {R, t}, where R is the rotation matrix, and t is the translation matrix. For example, $T_{WC}$ represents the transformation from the checkerboard to the color camera coordinate system, and a point $X_{W}$ in {W} can be transformed into {C} by the equation $X_{C}=R_{WC}X_{W}+t_{WC}$.

The above formulas can achieve the conversion of most coordinate systems, such as $T_{WC},\;T_{WE}$ and $T_{VD}$, but they cannot describe the relationship between {D} and {C}. Here, we use Herrera’s [18] method. Since the calibration plate ({V}) and the checkerboard ({W}) have coplanar characteristics, and $T_{WC},\;T_{VD}$ are known. Hence, we can get $T_{DC}$. Specific steps are as follows, and we define a plane with Formula (10) in each reference frames ({W}, {V}):

(10) $$n^{T}X-\delta =0$$

where **n** is the unit normal and δ is the distance to the origin. In addition, if the rotation matrix is defined as $R=\left(r_{1},r_{2},r_{3}\right)$, and the parameters of the plane in both frames are chosen as $n={\left[0,0,1\right]}^{T}$ and δ=0, then the plane parameters in the color camera coordinate system ({C}) are

(11) $$n=r_{3}\;\mathrm{and}\;\delta =r_{3}^{T}t$$

where it can use $R_{WC}$, $t_{WC}$ for the color camera and $R_{VD}$, $t_{VD}$ for the depth camera [18,20]. 

The plane parameters’ vectors for each color image could be concatenated by the matrices: $M_{C}=\left[n_{c1},n_{c2},\cdot \cdot \cdot ,n_{cn}\right]$ and $b_{C}=\left[\delta_{c1},\delta_{c2},\cdot \cdot \cdot ,\delta_{cn}\right]$ [28]. Furthermore, the plane parameters vectors in the depth camera could also be represented by $M_{D}$ and $b_{D}$. Then, the relative transformation $T_{CD}=\left\{R_{CD},t_{CD}\right\}$ is shown as:

(12) $$R\prime_{CD}=M_{D}M_{C}^{T}$$

(13) $$t_{CD}={\left(M_{C}M_{C}^{T}\right)}^{-1}M_{C}{\left(b_{C}-b_{D}\right)}^{T}$$

Finally, the rotation matrix $R_{CD}={\mathrm{UV}}^{T}$ is obtained by singular value decomposition (SVD), where ${\mathrm{USV}}^{T}$ is the SVD of $R\prime_{CD}$. $T_{DC}$ can also be obtained by $T_{CD}$. Now, the relative position between the three external cameras and the color camera of Kinect can be obtained directly.

### 3.3. Nonlinear Minimization

Least square method is a basic, practical, and widely used mathematical model [29], by minimizing the sum of squares of the error between samples and its reconstruct samples to find the best cost function. During the camera calibration, the core of the calibration method aims to minimize the weighted sum of squares of the measurement re-projection errors over all parameters. The re-projection error for the color camera and external camera are the Euclidean distance between the measured corner position and its re-projected position. We assume that the re-projection positions of the color camera and the external camera are ${\hat{p}}_{c}$, ${\hat{p}}_{e}$, respectively, and their actual measurement positions are $p_{c}$, $p_{e}$, respectively. For the depth camera, the re-projection error is the difference between the original disparity measurement value d and the re-projection value $\hat{d}$ (i.e., the estimated value of the original disparity) of the disparity. In Formula (4), $c_{0}$ and $c_{1}$ are the internal parameters of the depth camera, $z_{k}$ can be obtained by the depth information, and then we can get the original disparity estimated value $\hat{d}$. The method of [30] can be used to obtain the parameter $Z_{kk}$ in Equation (5), and the original disparity measurement value d can also be obtained. At this point, we have a preliminary cost function:

(14) $$c=\frac{\Sigma {\left\|{\hat{p}}_{c}-p_{c}\right\|}^{2}}{\sigma_{c}^{2}}+\frac{\Sigma {\left(\hat{d}-d\right)}^{2}}{\sigma_{d}^{2}}+\frac{\Sigma {\left\|{\hat{p}}_{e}-p_{e}\right\|}^{2}}{\sigma_{e}^{2}}$$

where $\sigma_{c}^{2}$, $\sigma_{d}^{2}$ and $\sigma_{e}^{2}$ are the variances of the measurement error of color camera, depth camera and external camera, respectively. Obviously, Formula (14) does not comply fully with our requirements. For example, some external camera parameters are completely not used. Hence, Equation (14) needs to be modified.

First of all, taking into account the disparity distortion correction of the depth camera, the estimated value $\hat{d}$ of the original disparity is replaced by ${\hat{d}}_{k}$ corrected by Equation (7). The measurement value d of original disparity is replaced by $d_{k}$ corrected by Equation (8). In Equation (8), the parameters $D_{\delta }$ and $\alpha =\left\{\alpha_{0},\alpha_{1}\right\}$ are independent from all of the other parameters. They only depend on the observed values of the pixel (u,v). Therefore, it can be optimized through least squares method individually, and the cost function of disparity distortion can be described as Equation (15). The initial values of $D_{\delta }$ and α are provided, and then the optimal solution by iteration is achieved:

(15) $$c_{d}=\underset{u,v}{\Sigma }\left({\hat{d}}_{k}-d_{k}\right)=\underset{u,v}{\Sigma }\left[\left(\frac{1}{c_{1}z_{k}}-\frac{c_{0}}{c_{1}}\right)-\left(d+D_{\delta }\left(u,v\right)\exp\left(\alpha_{0}-\alpha_{1}d\right)\right)\right]$$

**Figure 5:**
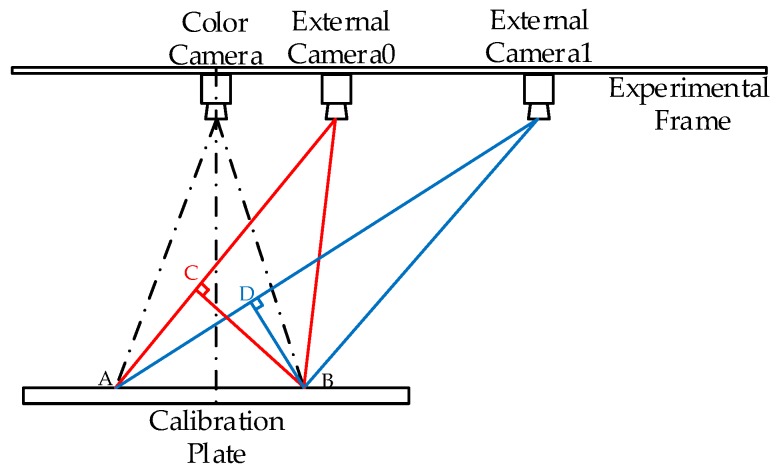
The top view of the experimental framework.

Secondly, the cost Function (14) cannot achieve the simultaneous calibration of all external cameras. On this basis, we extend the intermediate term in Equation (14) that is associated with external cameras. Meanwhile, adding different weights to the external cameras, that is, adding coefficients $\beta_{i}$ (i=0, 1, 2, 3 ...) to their corresponding re-projection errors. It can be found that the additional weights are related to the distance from the external cameras to the Kinect. Figure 5 shows the top view of the experimental framework during the image acquisition process. There are multiple rotation direction of the checkerboard plane, and the frontal plane is selected as the analysis object. The distance between points A and B is the total width of the checkerboard, and the distance between points B, C and points B, D are the width of the checkerboard shown in the pictures, which is taken by the external camera 0 and 1, respectively. Apparently, the distance between points B and C is longer than the distance between points B and D [31]. In other words, under the same condition, the checkerboard area occupies more pixels in the picture taken by the external camera 0. That is, the pictures that are taken by the external camera 0 contain more calibration information [32]. Therefore, it is believed that it should have a higher weight. That is to say, in the calibration process, when attaching a high weight to the camera0 that comes closer to the Kinect, and attaching low weights to camera1 and camera2 that are far from the Kinect, the calibration results are more accurate. 

This paper uses **I** to represent the spatial distance between the color camera and the corresponding external cameras on the experimental frame. By analyzing a large number of calibration results, the relationship between the spatial distance **I** and the correspondence coefficient β can be summed up. When the value of **I** for all of the external cameras is less than 600 mm, the value of coefficients β does not vary with **I**, and $\beta_{i}=1$; when the value of **I** for one or more external cameras is greater than 600 mm, it can be defined that A=(I−600)/50, β=1−0.02×A, and *A* is a natural number (e.g., 1.1 calculated as 2). At the same time, in order to reduce the influence of the external cameras on Kinect internal parameters calibration, we specify $\beta_{0}+\beta_{1}+...+\beta_{i}=i+1$ [33], and the other external cameras for which the value of I is less than 600 mm have the same value of coefficient; when the value of **I** for all of the external cameras is greater than 600 mm, all the external cameras coefficients are processed according to the same formula A=(I−600)/50, β=1−0.02×A. In this paper, the relative position between each corresponding external cameras and color camera can been calculated, and the corresponding external cameras coefficients as shown in Table 1. After analysis of the external cameras, the modified optimized cost function can also be obtained:

(16) $$c=\frac{\Sigma {\left\|{\hat{p}}_{c}-p_{c}\right\|}^{2}}{\sigma_{c}^{2}}+\frac{\Sigma {\left({\hat{d}}_{k}-d_{k}\right)}^{2}}{\sigma_{d}^{2}}+\beta_{i}\frac{\Sigma {\left\|{\hat{p}}_{ei}-p_{ei}\right\|}^{2}}{\sigma_{ei}^{2}},\;(i=0,1,2,3...)$$

It is easy to see that Formula (15) is the same as the corresponding intermediate term of the new cost Function (16) and does not interact with the others parameters. Therefore, we can directly replace the corresponding initial value in Equation (16). The nonlinear minimization of the parameters can be achieved by iteratively calculating the new cost function. The specific iteration process is as follows: the first step is to keep $D_{\delta }$ as a constant while assigning the coefficients $\beta_{0}$, $\beta_{1}$ and $\beta_{2}$ by 1.2, 0.9 and 0.9, respectively. Then, all the other parameters are substituted into Equation (16) to minimize the value of *c*. In the second step, the initial values of $\alpha_{0}$, $\alpha_{1}$ and $D_{\delta }$ in the depth distortion model are assigned to zero, and then they are taken into Equation (15) to optimize the disparity distortion parameter $D_{\delta }$ for each pixel individually. Once the new value $D_{\delta }$ is obtained, the old value $D_{\delta }$ is replaced in the first step. Repeat Steps 1 and 2 as many times as necessary until the residuals converge to a minimum.

## 4. Experiments

In order to demonstrate the performance of the proposed method in the real project, all of the input images in this experiment come from the same database, which were collected and produced by our existing experimental equipment. All pictures were collected in the way described in Section 3.1 and saved in JPG format. For comparison with Herrera’s method, all depth images in this experiment are saved in the same PGM format as in Herrera’s method. In addition, since Herrera’s method had a strong dependency on the number of input pictures, the results were random when the number of pictures was less than 20 [19], and the joint calibration method proposed in this paper only needs 15 pictures. The devices’ intrinsic parameters calculated by our method are shown in Table 2 and Table 3, wherein C.C. represents Color Camera and E.C. represents External Camera.

### 4.1. Herrera’s Method Results for Comparison

In our results, each device corresponds to a unique set of values. In this paper, the Herrera’s method results are used to compare with the proposed method. However, Herrera’s calibration method is limited to a single external camera and could not be effectively employed in multiple devices. We can only calibrate each external camera one by one. Therefore, in the actual calibration process, each of the different external cameras will correspond to a new set of Kinect data. How to choose from multiple sets of Kinect parameters is also a problem. In the actual comparison process, Herrera’s method is still used to calibrate the external camera 0, 1, 2, and there are three different sets of Kinect parameter values.

In the process of selecting Kinect parameters for Herrera’s method, the re-projection error value of color camera and depth camera is an important reference, the smaller the value is, the greater the selectivity of this set of Kinect parameters will be. Then, we select the single set of Kinect parameters, based on which the lowest re-projection error summed over all three external cameras is calculated. In addition, we can also put each set of Kinect parameter values into the 3D reconstruction module, respectively. By observing the effect of 3D reconstruction, the best group of values for Herrera’s method is chosen. However, the randomness of this method is too large, and the choice of Kinect parameters may be affected by the observation error. Therefore, this paper selects the Kinect parameters by the first method described above.

**Figure 6:**
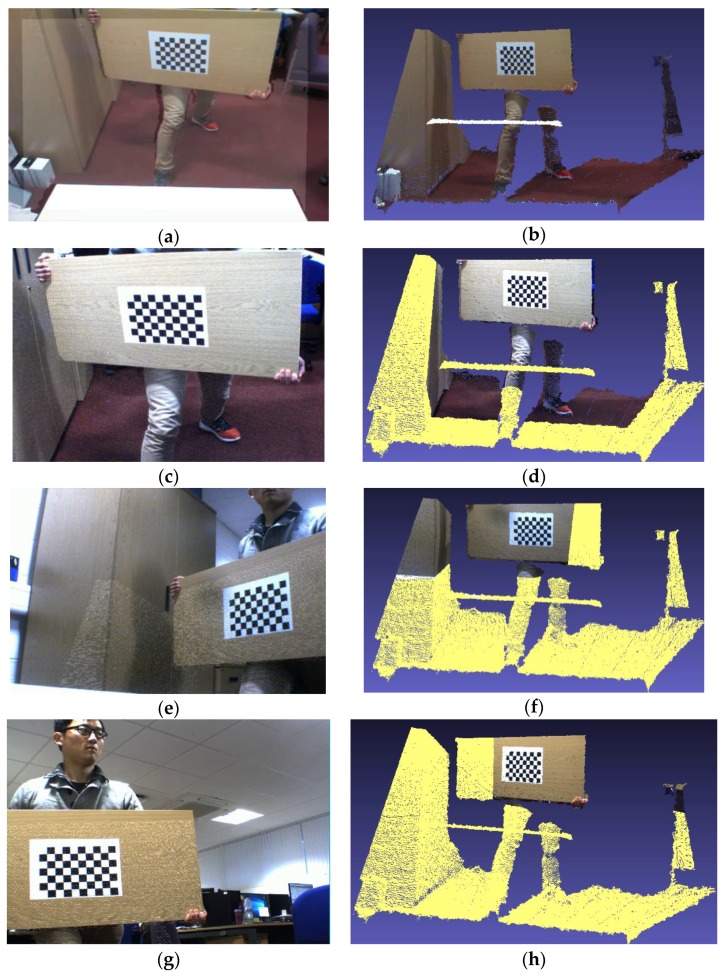
The overlaid depth maps and the corresponding 3D colored point cloud images obtained by proposed method. (**a**) the color image is captured by the color camera in Kinect; (**c**,**e**,**g**) the color images are captured by the external camera 0, 1 and 2, respectively; (**b**,**d**,**f**,**h**) are the corresponding 3D colored point cloud images, and they are captured from (**a**,**c**,**e**,**g**), respectively.

**Figure 7:**
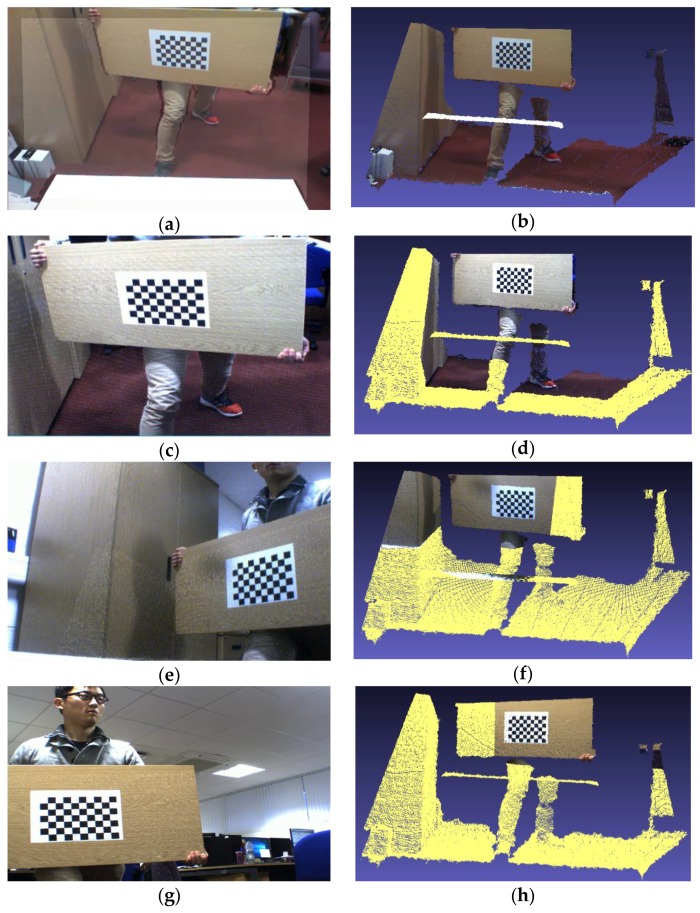
The overlaid depth maps and the corresponding 3D colored point cloud images obtained by Herrera’s method. (**a**) the color image is captured by the color camera in Kinect; (**c**,**e**,**g**) the color images are captured by the external camera 0, 1 and 2, respectively; (**b**,**d**,**f**,**h**) are the corresponding 3D colored point cloud images, they are captured from (**a**,**c**,**e**,**g**), respectively.

In order to visually present the difference between the two methods, in this paper, the corresponding rotation, translation and distortion correction are made to the original depth maps, and overlaid it on the corresponding color image [34]. The overlaid depth maps and the corresponding 3D colored point cloud images obtained by the proposed method and Herrera’s method are shown in Figure 6 and Figure 7, respectively. 

It can be clearly observed that the proposed method shows very accurate results in the corresponding overlaid depth maps and 3D colored point cloud images. Herrera’s method only satisfies partial accuracy in the corresponding overlaid depth maps and 3D colored point cloud images. For example, in Figure 7f, a large black point cloud appears on the white desktop, which is not allowed.

By analyzing the calibration results of the two methods for the same dataset, standard deviation is compared for the re-projection error as shown in Table 4. Here, the standard deviation of each re-projection error can be regarded as the actual value of the corresponding intermediate term after the nonlinear minimization by Formula (16). Therefore, the actual value of c in Equation (16) indirectly reflects the accuracy of the calibration, and it can be a reference to evaluate the accuracy of calibration, but it is by no means a direct standard [35]. The smaller the value is, the higher the calibration accuracy of the corresponding device will become. In Herrera’s method, the minimum value of the standard deviation of the color camera and the depth camera is found to be 0.1272 and 0.7343, respectively, and the standard deviation of the three external cameras is unique. This moment, the actual value of c is $c_{Her}=6.04436$ by Herrera’s method. Similarly, the c value of proposed method is $c_{Pro}=5.93022$. It can be found intuitively that the results of these two methods are very close. Both methods achieve accurate calibration, and the data shows that the proposed joint calibration method is more accurate. Therefore, our method does not only realize the joint calibration of the depth sensor and multiple external cameras, but also improves the accuracy of calibration and reduces the dependence on the number of input images.

### 4.2. 3D Reconstruction

**Figure 8:**
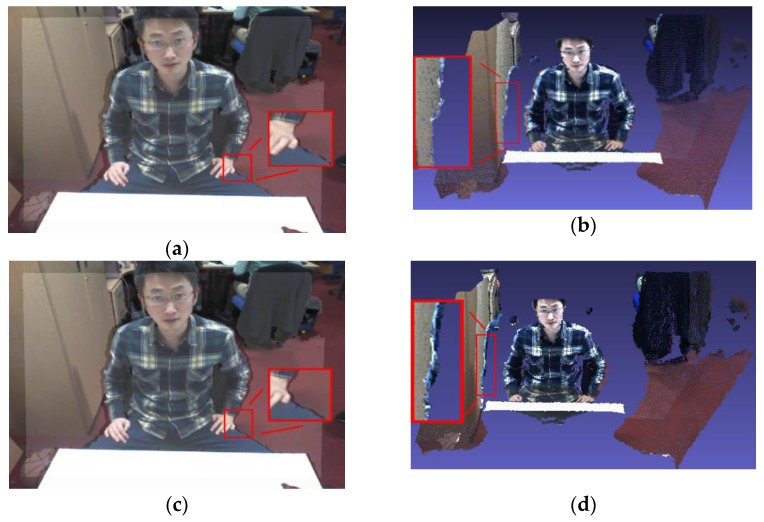
Joint 3D reconstruction. (**a**) and (**b**) are the overlaid depth map and the corresponding joint reconstructed 3D image by proposed method, respectively; (**c**,**d**) are the overlaid depth map and the corresponding joint reconstructed 3D image by Herrera’s method, respectively.

In addition, in order to provide data support for the 3D reconstruction module, the results of the two methods are also implemented into a real project platform, respectively [36]. The overlaid depth maps and the corresponding joint 3D reconstruction results of the proposed method and Herrera’s method are shown in Figure 8. Color images captured in different cameras are superimposed on the same space. The color images view comes from the color camera in Kinect and the external camera 0, which are covered in the same 3D point cloud space. The completeness of the reconstruction between them can reflect the accuracy of the joint calibration results. By observing the effects of the overlaid depth maps and the corresponding joint reconstructed 3D images, it can be found that both of these two methods ensure the integrity of the depth information, and the details of the scene are also reflected in the reconstructed 3D images. Comparing the details of these two image sets, the proposed method works better on the overlaid depth maps and the corresponding joint reconstructed 3D images. For example, in Figure 8a,c, comparing the left palm edge of the observed object, it is clear that the color and depth information were superimposed more accurately by proposed method; in Figure 8b,d, comparing the right shoulder of the observed object, the contours by the proposed method are clearer. In Herrera’s method, it contains a larger area of the clothing pattern on the surface of the brown storage locker due to the data deviation.

### 4.3. 3D Ground Truth

**Figure 9:**
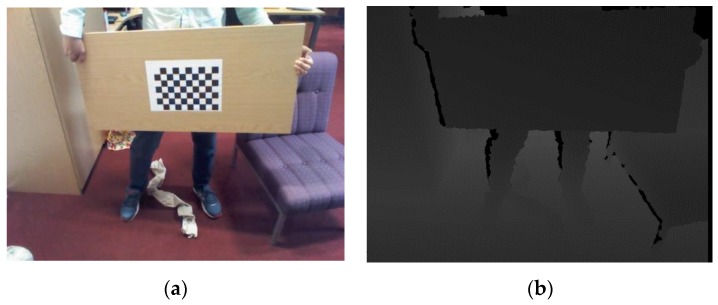
One of the test data sets. (**a**) the checkerboard image under Kinect view; (**b**) the corresponding depth image.

In order to visually demonstrate the calibration result of the two methods, we also collected a set of data as a test set. As shown in Figure 9, the test set contains six sets of standard chessboard images with different angles, and each set of images contains a checkerboard image under Kinect view and a corresponding depth image. First of all, the coordinates of the checkerboard corners of the test set are determined, and the number is in Figure 3a. The actual distance between the corners of the checkerboard is 25 mm. Then, the Kinect intrinsics of these two methods are used to reconstruct the test set, respectively. The calibration accuracy is evaluated by analyzing the distance error between the reconstructed points. In theory, the closer the actual distance and the calculated distance of the adjacent checkerboard corners are, the higher the calibration accuracy of the corresponding calibration method will be [37]. In order to reduce the relative error, the maximum known distances of the *x*-axis and the *y*-axis are measured separately. In other words, the distances between the checkerboard corners numbered 1, 9 and 1, 46 are calculated, respectively. Table 5 shows the distance error between the reconstructed points in the *x*-axis and *y*-axis directions. It is clear that the proposed method is closer to the true distance with a higher calibration accuracy.

## 5. Conclusions

Considering the problem that current research only focuses on the calibration of a single external camera instead of multiple external cameras, we present a novel method and a corresponding workflow framework that can simultaneously calibrate relative poses of a Kinect and three external cameras. By optimizing the final cost function and adding corresponding weights to the external cameras in different locations, the joint calibration of multiple devices is efficiently constructed. At the same time, the validity and accuracy of the method are verified with comparative experiments. Experimental results show that the proposed method improves the accuracy of calibration. It also shows that the proposed method does not only reduce the dependence on the number of input pictures, but also improves the accuracy of joint 3D reconstruction. In this paper, camera calibration technology is used to provide data support and has been successfully applied in a practical real-time project, with important practical value.

## Figures and Tables

**Table 1:** External camera correspondence coefficient.

	X	Y	Z	I	β
E.C.0	71.58	39.91	33.03	88.36	1.2
E.C.1	482.43	585.62	346.42	834.04	0.9
E.C.2	−505.84	569.28	370.63	846.95	0.9

**X**, **Y** and **Z** are the corresponding coordinates in the experimental frame coordinate system, respectively; **I** is the spatial distance of the color camera and the corresponding external camera in the coordinate system; β is the corresponding coefficient of the external camera.

**Table 2:** Color camera intrinsic parameters.

	$f_{\mathrm{cx}}$	$f_{\mathrm{cy}}$	$u_{c0}$	$v_{c0}$	$k_{c1}$	$k_{c2}$	$p_{c1}$	$p_{c2}$	$k_{c3}$
C.C.	518.52	520.68	324.31	243.74	−0.0124	0.2196	0.0014	−0.0003	−0.5497
	±0.07	±0.06	±0.10	±0.10	±0.0016	±0.0225	±0.0001	±0.0001	±0.0995
E.C.0	1619.83	1626.44	633.85	475.47	−0.0540	−3.1424	−0.0011	−0.0034	7.4728
	±4.66	±4.95	±15.13	±21.08	±0.1706	±4.1635	±0.0025	±0.0020	±2.7466
E.C.1	1652.77	1652.86	695.53	477.55	−0.3491	0.4026	−0.0004	0.0047	6.4104
	±8.04	±8.06	±15.22	±13.44	±0.1422	±2.5525	±0.0017	±0.0018	±4.2151
E.C.2	1638.09	1637.34	766.41	503.78	0.1555	0.8307	−0.0049	0.0120	−2.1837
	±7.15	±7.88	±18.46	±15.55	±0.0635	±0.7039	±0.0013	±0.0025	±2.2819

This table shows the focal length $\left(f_{cx},f_{cy}\right)$, the principal point ($u_{c0}$, $v_{c0}$) and the distortion coefficient $K_{c}=$ [$k_{c1}$ $k_{c2}$ $p_{c1}$ $p_{c2}$ $k_{c3}$], respectively, wherein C.C. and E.C. represents Color and External Camera, respectively.

**Table 3:** Depth sensor intrinsic parameters.

$f_{\mathrm{dx}}$	$f_{\mathrm{dy}}$	$u_{d0}$	$v_{d0}$	$k_{d1}$	$k_{d2}$	$p_{d1}$
573.87	573.13	327.10	234.93	0.0487	0.0487	−0.0035
±0.00	±0.00	±0.00	±0.00	±0.0000	±0.0000	±0.0000
$p_{d2}$	$k_{d3}$	$c_{0}$	$c_{1}$	$\alpha_{0}$	$\alpha_{1}$	
−0.0042	0.0000	3.42	−0.003162	0.8656	0.0018	
±0.0000	±0.0000	±0.001457	±0.00	±0.0460	±0.0001	

This table shows the focal length ($f_{dx}$, $f_{dy}$), the principal point ($u_{d0}$, $v_{0}$), the distortion coefficient $K_{d}=$ [$k_{d1}$ $k_{d2}$ $p_{d1}$ $p_{d2}$ $k_{d3}$], the depth parameters ($c_{0}$, $c_{1}$) and the depth distortion ($\alpha_{0}$, $\alpha_{1}$), respectively.

**Table 4:** Standard deviation of re-projection error.

	Herrera’s Method			Proposed Method
**C.C.**	0.1367	**0.1272**	0.1390	0.1423
	[−0.0054, +0.0058]	[−0.0050, +0.0054]	[−0.0055, +0.0059]	[−0.0056, +0.0061]
**E.C.0**	1.7242			**1.6984**
	[−0.0658, +0.0709]			[−0.0648, +0.0699]
**E.C.1**		1.8169		**1.6580**
		[−0.0739, +0.0801]		[−0.0674, +0.0731]
**E.C.2**			1.6429	**1.5566**
			[−0.0646, +0.0699]	[−0.0612, +0.0662]
**D.C.**	0.8455	**0.7343**	0.7829	0.8567
	[−0.0012, +0.0012]	[−0.0010, +0.0010]	[−0.0011, +0.0011]	[−0.0011, +0.0012]
c	6.04436			**5.93022**

Wherein C.C. represents Color Camera; E.C. represents External Camera and D.C. represents Depth Camera; c is the parameter in Equation (16). To compare the data sets, the variances were kept constant ($\sigma_{c}=0.02$ px, $\sigma_{d}=0.75$ kud, $\sigma_{ei}=0.40$ px).

**Table 5:** Distance errors between the reconstructed points.

	Herrera’s Method		Proposed Method	
	Lx-25 (mm)	Ly-25 (mm)	Lx-25 (mm)	Ly-25 (mm)
1	0.16988	0.07660	0.16475	0.06380
2	0.10438	0.10360	0.09775	0.09040
3	0.19263	0.08220	0.18025	0.06960
4	0.20350	0.25660	0.18088	0.24160
5	−0.05288	0.20440	−0.04725	0.19200
6	0.03600	0.07500	0.03288	0.06520
M	0.12655	0.13307	**0.11729**	**0.12043**

Lx and Ly represent the calculated distances of the adjacent checkerboard corners in the *x*-axis and *y*-axis directions, respectively. Lx-25 and Ly-25 represent the error between the calculated distance and the actual distance in the *x*-axis and *y*-axis directions, respectively. M represents the arithmetic mean of the absolute value of the distance error.
